# Supplementary material for: Content-rich biological network constructed by mining PubMed abstracts
Source: BMC Bioinformatics. 2004 Oct 8;5:147. doi: 10.1186/1471-2105-5-147 (PMC528731; doi:10.1186/1471-2105-5-147)
Supplement: Additional File 2 — The original results of the above study (non-essential files are deleted to keep the file size under the limit set by BMC bioinformatics). [file 1471-2105-5-147-S2.bz2 › chilibotAdditionalFile2/dip20/235ID8006584E759/html/IL2RB_IL2.html]

 


 **IL2RB** and **IL2** 
  
Found 3819 abstracts in PubMed, retrieved 20.  
 

 What does Google say? 
 PDF only 
| .edu only 

---

**Interactive relationship** (e.g. stimulation, inhibition, etc)

**Neutral relationship**- BACKGROUND Daclizumab 150 kD, a humanized monoclonal antibody mAb against the alpha chain of the membrane bound interleukin 2  [ **IL2** ]  IL 2  [ **IL2** ]  receptor  [ **IL2RB** ]  IL 2R also binds soluble interleukin 2R alpha sIL 2Ralpha.  Ref: 12846768 Kidney Int, 2003

**Non-interactive relationship** (e.g. studied together, co-existance, homology, etc.)

- CONCLUSION The monoclonal antibody against IL 2  [ **IL2** ]  receptor  [ **IL2RB** ]  alpha chain CD 25 has a marked effect upon severe steroid refractory GVHD.  Ref: 12812665 Zhonghua Yi Xue Za Zhi, 2003
- Immunosuppression protocol anti IL 2  [ **IL2** ]  receptor  [ **IL2RB** ]  antibodies, steroids, mycophenolate mofetil MMF.  Ref: 12876872 Acta Med Croatica, 2003
- Addition of IL 12 to this coculture prevented apoptosis of the CD4 T cells, which was associated with up modulation of IL 2  [ **IL2** ]  receptor  [ **IL2RB** ]  beta chain expression.  Ref: 12883484 Hepatology, 2003
- The suppressive activity of CD8 CD25 thymocytes was abrogated by a mixture of anti CTLA 4 and anti TGF beta1 antibodies and it was mediated by their ability to inhibit the expression of the IL 2  [ **IL2** ]  receptor  [ **IL2RB** ]  alpha chain on target T cells.  Ref: 12893750 BloodBlood, 2003
- The inhibitory effect on human T cells was assessed and demonstrated at the level of proliferation DNA synthesis, IL 2  [ **IL2** ]  secretion, and IL 2  [ **IL2** ]  receptor  [ **IL2RB** ]  CD25 upregulation.  Ref: 12835898 Inflamm Res, 2003
- Although the effects of mitogens on the synthesis of interleukin 2  [ **IL2** ]  IL 2  [ **IL2** ]  and IL 2  [ **IL2** ]  receptor  [ **IL2RB** ]  IL 2r have been described, a detailed in situ analysis of the spatio temporal changes of the expression of the IL 2  [ **IL2** ]  gene and the three IL 2r components in lymphoid tissues is still missing.  Ref: 12890448 Cytokine, 2003
- Most investigated Treg cells are CD4 pos cells expressing the alpha chain of the IL 2  [ **IL2** ]  receptor  [ **IL2RB** ]  CD25.  Ref: 12848995 Autoimmun Rev, 2002
- Plasticity of IL 2  [ **IL2** ]  and IL 2  [ **IL2** ]  receptor  [ **IL2RB** ]  chains in rat lymphoid tissues in situ after stimulation with staphylococcal enterotoxin A.  Ref: 12890448 Cytokine, 2003
- The pathological changes of the graft, IL 2  [ **IL2** ]  receptor  [ **IL2RB** ]  expression in lamina propria lymphocytes, serum IL 2  [ **IL2** ]  concentrations, results of spleen lymphocytes transformation test and the IL 2  [ **IL2** ]  secretion capacity were determined and compared.  Ref: 12875685 Chin Med J (Engl), 2003
- Abnormal IL 2  [ **IL2** ]  receptor  [ **IL2RB** ]  levels in non pregnant women with a history of recurrent miscarriage.  Ref: 12832383 Hum Reprod, 2003
- The concentration of soluble IL 2  [ **IL2** ]  receptor  [ **IL2RB** ]  R protein was measured in BAL fluid using ELISA as well, as an indicator of lymphocyte activity.  Ref: 12869037 Clin Exp Immunol, 2003
- When we compared mean nocturnal levels, CD16 and CD25 T and B activated lymphocytes with expression of the a chain of IL 2  [ **IL2** ]  receptor  [ **IL2RB** ]  levels were higher, while CD8, CD8bright, CD20 total B cells, TcRd1 epitope of the constant domain of d chain of T cell receptor 1 and dTcS1 epitope of the variable domain of d chain of T cell receptor1 levels were lower in cancer patients.  Ref: 12797908 Int J Immunopathol Pharmacol< MedlineTA>Int J Immunopathol Pharmacol, 2003
- There was no significant expression of IL 2  [ **IL2** ] , IL 2  [ **IL2** ]  receptor  [ **IL2RB** ] , IL 4, TNFalpha, or IFNgamma.  Ref: 12826912 Pancreas, 2003
- Anti lymphocyte antibodies ATG deplete circulating lymphocytes while selective monoclonal antibodies are directed against IL 2  [ **IL2** ]  receptor  [ **IL2RB** ]  thus reducing the rate of proliferation of activated T cells.  Ref: 12835079 Mol Immunol, 2003
- We detected an upregulation or de novo synthesis of interleukin 1alpha IL 1alpha, IL 1beta, IL 8, macrophage inflammatory protein 1alpha MIP 1alpha, MIP 1beta, GRO alpha, transforming growth factor beta, MCP 1, IL 2  [ **IL2** ]  receptor  [ **IL2RB** ]  gamma IL 2Rgamma, IL 10Ralpha, and IL 6R.  Ref: 12874345 Infect Immun, 2003
- METHODS Thrombin antithrombin III complex TAT, plasma D dimer, serum type III procollagen peptide PIIIP, serum soluble IL 2  [ **IL2** ]  receptor  [ **IL2RB** ]  sIL 2R, Free tissue factor pathway inhibitor TFPI, and soluble intercellular adhesion molecule ICAM 1 were measured preoperatively around the same period when computed tomography CT was taken in 17 patients with AA mean age 72.2 years.  Ref: 12829084 Eur J Cardiothorac Surg, 2003
- In this study, the authors observed that combined use of IL 18 and low dose IL 2  [ **IL2** ]  synergistically promoted in vitro proliferation of natural killer cells with up regulation of IL 2  [ **IL2** ]  receptor  [ **IL2RB** ]  alpha and also synergistically stimulated cytolytic activity and interferon gamma production by these cells.  Ref: 12806277 J Immunother< MedlineTA>J Immunother, 2003
- Presentation by these migrants sustained expression of the IL 2  [ **IL2** ]  receptor  [ **IL2RB** ]  and promoted delayed type hypersensitivity.  Ref: 12871638 Immunity, 2003
- Serum LDH and soluble IL 2  [ **IL2** ]  receptor  [ **IL2RB** ]  were high in titers 552 U l and 90 U l, normal range 145 519.  Ref: 12820543 Rinsho Shinkeigaku< MedlineTA>Rinsho Shinkeigaku, 2003
- We found a defective T cell response in malnourished children, as indicated by diminished levels of circulating total CD3, helper CD4, IL 2  [ **IL2** ]  receptor  [ **IL2RB** ]  bearing CD4 CD25 and memory helper T cell responses CD4 CD45RO in keeping with the decreased specific IgE levels against Ascaris lumbricoides.  Ref: 12848201 J Trop Pediatr, 2003
